# Percutaneous Cholecystostomy for Acute Cholecystitis: A Three-Year Single-Centre Experience Including During COVID-19

**DOI:** 10.7759/cureus.20385

**Published:** 2021-12-13

**Authors:** Taner Shakir, Kabir Matwala, Abhilash Vasan, Stavros Karamanakos

**Affiliations:** 1 General Surgery, Mid and South Essex NHS Foundation Trust, Basildon, GBR; 2 General and Colorectal Surgery, Mid and South Essex NHS Foundation Trust, Basildon, GBR

**Keywords:** emergency surgery, covid-19, cholecystectomy, cholecystitis, cholecystostomy

## Abstract

Introduction

Percutaneous cholecystostomy is a recognised treatment modality for acute cholecystitis. Traditionally, its use was reserved for patients deemed unfit for surgery. However, the coronavirus disease 2019 (COVID-19) pandemic had a detrimental effect on both elective and emergency surgery. The utilisation of cholecystostomy thus increased. Unanswered questions remain over timing with respect to interval cholecystectomy. We evaluated our local practice over the preceding three years.

Methods

A retrospective analysis was performed of all patients who had a percutaneous cholecystostomy inserted over a three-year period (1 January 2018-1 January 2021). The primary outcome was time to cholecystectomy. Secondary outcomes were cholecystostomy-related complications, 30-day mortality, cholecystectomy-related complications and length of postoperative hospital stay.

Results

A total of 31 patients were identified during the period. Thirteen (42%) patients went on to have a laparoscopic cholecystectomy. The median time interval from cholecystostomy to cholecystectomy was 97 days (interquartile range [IQR]: 81-140, minimum: 47 and maximum: 791). One case was complicated by small bowel perforation; this occurred after an interval of 106 days. The median length of postoperative stay was one day (IQR: 1-1, minimum: 0 and maximum: 4). Cholecystostomy-related complications were observed in four (13%) patients, whereby three became displaced and one developed blockage. Thirty-day mortality following cholecystostomy insertion was zero.

Conclusions

Percutaneous cholecystostomy is a safe and effective intervention for the management of acute cholecystitis. Interval cholecystectomy should be carefully considered; it may be safer to perform prior to 90 days.

## Introduction

Acute cholecystitis is one of the most common emergency presentations on the general surgical take. Its prevalence in the United Kingdom (UK) has been estimated to be 19% in males and 29% in females [1]. Management differs between institutions, varying between conservative and emergency surgical treatment depending on local facilities and expertise. National Institute for Health and Care Excellence (NICE) guidance recommends early laparoscopic cholecystectomy within one week of a diagnosis of cholecystitis [2]. Furthermore, NICE guidelines recommend elective laparoscopic cholecystectomy to be offered to all patients following percutaneous cholecystostomy when well enough for surgery [2]. Nonetheless, In a UK-based cohort study, 16.7% of patients were found to have had an emergency cholecystectomy, whereas elective and delayed cholecystectomy comprised 83.8% of the cohort [3].

However, the coronavirus disease 2019 (COVID-19) pandemic had a deleterious impact on both elective and emergent surgery. The first national lockdown was imposed in the UK on 23 March 2020 and lasted until a phased reopening throughout June 2020 [4]. A second lockdown was from the period of 5 November to 2 December 2020. A subsequent third lockdown was then aimed at controlling the spread of variant strains and lasted from 5 January 2021 to 8 March 2021, and gradually lifted over the coming months. An estimated 28 million elective operations were cancelled globally [5]. Healthcare strategies adapted to focus on ambulatory methods of care [6]. Guidelines from intercollegiate associations in the UK recommended the avoidance of operative management, or when necessary, to perform open surgery [7].

With the associated morbidity of an open cholecystectomy, a trend towards radiological drainage was observed. Percutaneous cholecystostomy is a radiologically sited drain to decompress the gallbladder and reduce the sepsis burden. Traditionally used in the setting of patients who were deemed unfit for surgery, its utilisation became commonplace with the strains placed on the healthcare system by the COVID-19 pandemic. Following cholecystostomy, optimal management of drains and subsequent interval cholecystectomy are uncertain topics. We retrospectively analysed the management in our district general hospital (DGH) over a three-year period.

## Materials and methods

Study design

The study aimed to ascertain whether there was an optimal time to perform interval cholecystectomy after insertion of percutaneous cholecystostomy. We retrospectively reviewed all percutaneous cholecystostomies performed by the interventional radiology department during the preceding three years. Patients with acute biliary pathology were included where drainage was performed in an emergent setting.

Setting

Our DGH offers services to a local population of approximately 1,200,000 residents in Middle and South Essex, located in the South East of the United Kingdom. It was one of the worst-hit areas in the country during the COVID-19 pandemic. The DGH benefits from a 24-hour on-call interventional radiology service.

Time Period

The study was conducted for three years from 1 January 2018 to 1 January 2021.

Outcomes

Primary Outcome

The primary outcome was the number of days from the insertion of percutaneous cholecystostomy to the patient having an interval cholecystectomy.

Secondary Outcomes

The secondary outcomes were cholecystostomy-related complications, cholecystectomy-related complications, length of postoperative hospital stay, 30-day mortality related to percutaneous cholecystostomy and cholecystectomy, respectively.

Data collection

The hospital's radiology system was queried for all patients who had a percutaneous cholecystostomy inserted by any means. Data were then sourced from patient pathway software, pathology and radiological reports, operating theatre management software, operation notes and anaesthetic charts. Data collected included patient demographics, length of stay (LOS), indication for cholecystostomy, method of cholecystostomy insertion, complications, 30-day mortality and date of subsequent cholangiogram. With regards to interval cholecystectomy, data included surgical approach, postoperative LOS, surgical complications and 30-day postoperative mortality during the study period. Complications were classified as per the Clavien-Dindo classification [8]. Data were collected and statistically analysed using Microsoft Excel (Microsoft Corporation, Redmond, WA) and independently checked and verified by two authors.

Ethical considerations

The local hospital ethics committee exempted this study from ethical approval and was registered as an audit.

## Results

Demographics

A total of 31 patients were identified during the study period. A total of 16 (52%) cases were performed prior to the COVID-19 and 15 (48%) were performed after the onset of the pandemic. The median age of the patients was 73 years (interquartile range [IQR]: 67-77). The male to female ratio was 1:0.9. The Charlson Comorbidity Index (CCI) was calculated for each patient; the median CCI was 5 (IQR: 2-7). The American Society of Anesthesiologists (ASA) grade was recorded in 18 patients with a median ASA of 3 (IQR: 2-3, minimum: 2 and maximum: 4). Body mass index (BMI) was listed in 19 (61%) patients, of these, the mean BMI was 29.

Cholecystostomy

All cases were performed under ultrasound guidance. The distribution of pathologies is listed below in Table 1. The median time interval from admission to insertion of cholecystostomy was three days (IQR: 2-4). Complications were observed in four (13%) patients, whereby three became displaced and one developed blockage. Three of these patients required further intervention with tube replacement. Interval T-tube cholangiograms were performed post-procedure in 19 (61%) patients. Median days from insertion to cholangiogram were 38 (IQR: 30-47) days. Thirty-day all-cause mortality was zero; however, two mortalities were observed at 52 and 58 days after insertion of cholecystostomy, respectively. Both these occurred after the onset of the COVID-19 pandemic.

**Table 1:** Indication for insertion of percutaneous cholecystostomy.

Indication (n = 31)	
Calculous cholecystitis	24
Acalculous cholecystitis	4
Empyema	1
Mucocele	1
Perforation	1

Cholecystectomy

Interval cholecystectomy was attempted in 13 (42%) patients. Demographic subgroup analyses of the operative versus non-operative groups are shown in Tables 2, 3. Eleven (85)% of these patients had their operation successfully completed laparoscopically. One (8%) patient required open conversion and the procedure was abandoned in one (8%) patient. Preoperatively, one patient was referred to a tertiary centre for further management. The median time interval from cholecystostomy to cholecystectomy was 97 days (IQR: 81-140, minimum: 47 and maximum: 791). Nine (75%) operations performed were by a specialist upper gastrointestinal surgeon. The median length of postoperative stay was one day (IQR: 1-1, minimum: 0 and maximum: 4). One (8%) complication of small bowel perforation was observed.

**Table 2:** Mean demographics of the operative subgroup. BMI, body mass index; ASA, American Society of Anesthesiologists; CCI, Charlson Comorbidity Index.

Operative (n = 14)			
Age	BMI	ASA	CCI
70	28	2	3

**Table 3:** Mean demographics of the non-operative subgroup. BMI, body mass index; ASA, American Society of Anesthesiologists; CCI, Charlson Comorbidity Index.

Non Operative (n = 17)				
Age	BMI	ASA	CCI	
75	29	3	6	

## Discussion

Cholecystostomy is an alternative treatment modality to conservative or surgical intervention in cholecystitis. Previously reserved for patients contraindicated for surgical management, the global pandemic resulted in a change in management. Ambiguities regarding viral transmission from surgical smoke plumes and evacuation of carbon dioxide gas post-laparoscopic procedures led to a trend towards non-surgical treatments [9]. Cholecystostomy was the celling of intervention during the COVID-19 pandemic. Almost half (48%) of cholecystostomies were performed in the final year of the three-year retrospective analysis. Analysing the monthly rate of cholecystostomy insertion, this almost doubled from 0.8 per month to 1.5 per month prior to and during COVID-19, respectively.

Demographically, patients tended to be poor candidates for surgery. The majority were aged over 65 years, with a BMI approaching clinically obese. These factors, in addition to the male sex, incorporate part of difficult cholecystectomy predictive scoring systems [10,11]. Furthermore, a CCI of over 6 was one factor associated with increased postoperative complications [12]. When correlated with ASA grade, this in part underlines the rationale behind the clinical decision-making with respect to radiological versus potential surgical management.

Cholecystostomy was a readily available intervention. Our DGH setting benefits from a 24-hour interventional radiology on-call service. The mode number of days until drainage was three. The minimum and the maximum number of days were zero and 10, respectively. The procedure was well tolerated with the most common complication being tube displacement. There were no major complications observed. Cholangiograms were well tolerated, with the majority performed approximately four to five weeks after tube insertion; this was delayed compared with the World Society of Emergency Surgery (WSES) guidelines of two to three weeks [13]. One patient required repeat cholangiograms due to a non-opacifying cystic duct. Three patients required re-intervention by means of re-siting their drain at the time of cholangiogram.

Delayed cholecystectomy was performed in 12 patients with calculus cholecystitis, and one with acalculous cholecystitis. The mean time till cholecystectomy was 116 days. Elective surgical intervention was reserved for younger, less co-morbid and lower BMI patients. This is highlighted in Tables 2, 3.

In patients undergoing operative intervention, all had attempted laparoscopic cholecystectomies. This was successful in 90% of the subgroup. Of the remaining two patients, one patient had the procedure abandoned due to dense adhesions, and one required conversion to open due to adhesions. More commonly seen after major abdominal surgery, adhesions can nonetheless develop in the inflammatory condition of cholecystitis. Coupled with the interventional procedure of cholecystostomy, the resultant fibrosis can make subsequent surgery more difficult. Indeed, the previous cholecystostomy is associated with increased postoperative morbidity [12]. Moreover, the one surgical complication observed was small bowel perforation, with dense intraoperative adhesions noted - a likely contributing factor.

Postoperatively, LOS was variable. Inpatient days ranged from zero to four. A linear regression model was used to ascertain if there was an impact of time between cholecystostomy and cholecystectomy, with postoperative LOS. There was no significant association between the two (R2 = 0.00034, p = 0.95). However, subgroup analysis highlighted a difference in mean LOS. Prior to 90 days and after 90 days from cholecystostomy, mean LOS were 0.67 and 1.57 days, respectively. In addition, the only surgical complication was observed in the latter group. This may indicate that a safer window to attempt operative intervention after insertion of cholecystostomy is prior to 90 days.

## Conclusions

Percutaneous cholecystostomy for the management of cholecystitis can be a safe and effective treatment modality. It is associated with low morbidity and may be an effective bridge to cholecystectomy. Elective cholecystectomy should be planned appropriately to avoid complications. Tertiary care referrals to hepatobiliary units may be required in complex cases. It may be safer to perform surgery prior to 90 days after cholecystostomy insertion.
